# Prevalence and Management of Incidental Testicular Masses—A Systematic Review

**DOI:** 10.3390/jcm11195770

**Published:** 2022-09-29

**Authors:** Daniel Henriques, Anabela Mota Pinto, Helena Donato, Ricardo Leão

**Affiliations:** 1 Faculdade de Medicina, Universidade de Coimbra, 3004-540 Coimbra, Portugal; 2 Instituto de Patologia Geral, Faculdade de Medicina, Universidade de Coimbra, 3004-504 Coimbra, Portugal; 3 Documentation and Scientific Information Service, Centro Hospitalar e Universitário de Coimbra, 3004-561 Coimbra, Portugal

**Keywords:** incidental findings, testicular neoplasms, ultrasonography, frozen sections, watchful waiting

## Abstract

Management of incidentally diagnosed small testicular masses (STM) is controversial. Although there is the risk of malignancy, it might be realistic to safely seek preservation of testicles bearing benign masses. This study aims to systematically evaluate the evidence regarding prevalence of STMs, their benign or malignant histology and their management. We conducted a systematic literature search for studies reporting small or incidental testicular masses and their management by radical orchiectomy, testis sparing surgery (TSS) or ultrasound (US) surveillance. We initially screened 2126 abstracts and from these, 57 studies met the inclusion criteria. Testicular masses were detected in 1.74% of patients undergoing US examination. Regarding STMs removed by surgery, 41.12% were benign. Intraoperative frozen section examination (FSE) is a reliable tool to discriminate between benign and malignant testicular masses (average 93.05% accuracy), supporting TSS. Benign lesions were associated with smaller diameter (<1 cm 68.78% benign), were often hypoechoic and exhibited regular margins on US. Conclusions: Small testicular masses are often benign. Clinical and US patterns are not accurate enough for including patients in surveillance protocols and TSS paired with FSE is pivotal for precluding the removal of testicles bearing benign lesions. Future research might unveil new imaging tools or biomarkers to support clinical management.

## 1. Introduction

Although relatively rare (1% of male neoplasms and 5% of urological tumors), testicular cancer (TC) is the most common malignancy in males aged 15–40 years with an increasing incidence during recent decades, particularly in industrialized countries [1,2,3].

Histologically, around 95–98% of all testicular cancers are testicular germ cell tumors (TGCT), which include seminomas (50–60% of tumors), nonseminomas (40–50%) and spermatocytic tumors (<1%). The remaining 5% are mostly sex cord-stromal tumors [4].

Clinically, testicular cancer presents, most frequently, as a palpable mass (TGCT in about 90% of the cases). Worryingly, the incidental identification of impalpable small testicular masses (STM) is increasingly frequent, most probably due to the widespread use of testicular ultrasonography (US) for other indications, particularly in the study of male infertility or testicular pain.

The approach to these nonpalpable STMs is classically an inguinal radical orchiectomy (RO), which might come in hand with side effects such as hypogonadism, infertility, sexual dysfunction and modified male body-image, which are particularly troublesome in the typically young testicular cancer survivors. In fact, RO took place all over the world despite reports of STMs being benign in a large percentage of cases [5,6]. Until 2019, RO was still considered the gold standard approach to testicular masses of unknown origin. Testicular sparing surgery (TSS) was considered an option only in special cases, such as in synchronous bilateral testicular tumors or tumor in a solitary testicle. However, it is now recommended to discuss TSS in patients with a high likelihood of having a benign testicular tumor suitable for enucleation [1].

All in all, in the last couple decades, TSS coupled with frozen section examination (FSE) is arising as a popular management option for patients with STMs.

Nonetheless, controversy remains in selecting the patients eligible for this conservative approach. Several factors may suggest an increased risk of developing a testicular cancer (e.g., age, tumor size, cryptorchidism, infertility, etc.) and therefore, can contribute to advising against TSS. Some complementary tools, such as US, might also allow for identification of malignant features of the mass (size, echogenicity, vascularization and calcifications).

Currently, there are no specific orientations for the management of these incidental lesions, which leads to divergent opinions amidst the medical professionals and disparate options in daily urological practice. As such, the aim of this review is to interrogate the current data concerning incidental STMs and provide evidence for their best management.

## 2. Materials and Methods

### 2.1. Protocol and Registration

This review was performed according to the Preferred Reporting Items for Systematic Reviews and Meta-analysis (PRISMA) statement [7]. The review protocol was published in PROSPERO database (https://www.crd.york.ac.uk/PROSPERO/display_record.php?RecordID=199322, (accessed on 1 August 2022); registration number CRD42020199322) (Supplementary Data—PROSPERO Protocol).

### 2.2. Information Sources/Search Strategy

In September 2020, we performed a systematic literature search using well-established international electronic databases: PubMed, EMBASE (via Elsevier), Cochrane Central Register of Controlled Trials-CENTRAL (via Wiley Online Library), Web of Science Core Collection (via Clarivate Analytics), ClinicalTrials.gov and MedRxiv. The search was conducted in the English language. A variety of terms related to key subject areas of the review questions were used. Keywords or database specific subject headings (e.g.: MeSH, and boolean operators (OR) and (AND)) were employed to combine search terms. The search terms were adjusted to the specificities of the different databases (Supplementary Data—Electronic search strategy). Search results in each database were scanned ranging from inception to November 2020. Additional end searches of the reference lists of all included studies were conducted to ensure completeness of the search.

### 2.3. Eligibility Criteria (PICO)

A study was considered relevant for this review if it addressed the following: Adults (>18 years) presenting with a testicular mass incidentally diagnosed by ultrasonography or otherwise small testicular masses treated surgically (P); Submitted to a conservative approach (surveillance), partial or radical orchiectomy surgery associated or not with frozen section (I); Malignant versus benign (O). Studies considered eligible were prospective cohort studies, randomized controlled trials, cross-sectional studies, case-control studies and case series. Studies in children, animal studies, tissue studies, single case reports, editorials, reviews and meeting abstracts were excluded. Studies with a low number of cases were individually reviewed and selected or excluded according to novelty and level of evidence.

### 2.4. Study Selection

**Figure 1:**
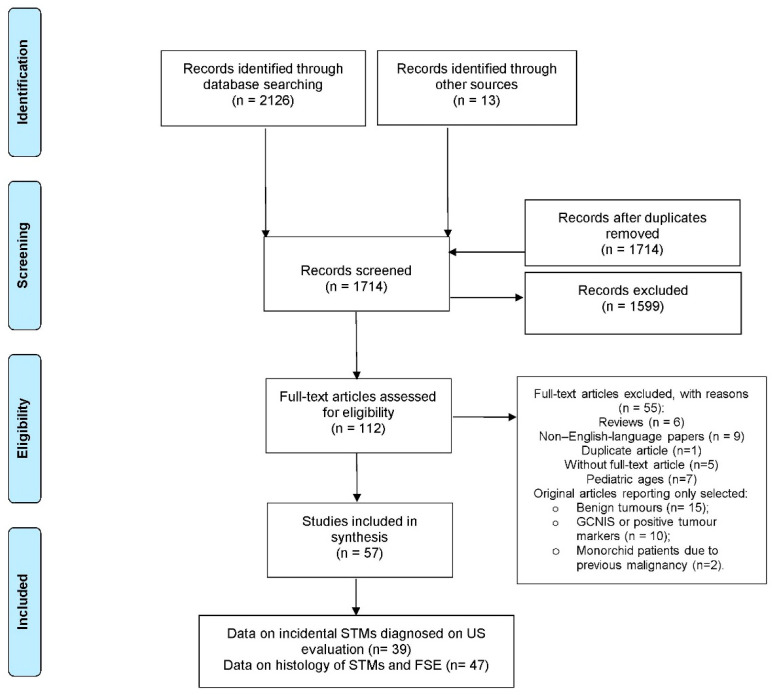
Flow diagram of evidence acquisition in a systematic review of studies addressing the prevalence and management of small testicular masses. GCNIS—germ cell neoplasia in situ; STMs—small testicular masses; US—ultrasound; FSE—frozen section examination.

All eligible articles were imported to and organized in the EndNote^®^ (Clarivate Analytics, Philadelphia, PA, USA) Web reference manager software. Duplicate publications were deleted automatically and then manually filtered. All authors participated in the design of the search strategy and in defining inclusion criteria. Two reviewers (DH, RL) screened all abstracts and full-text articles independently. Disagreement was resolved by discussion among the panel of co-authors. The final list of included manuscripts was selected with the consensus of all collaborators. The PRISMA flow diagram documented included and excluded studies and the reasons for exclusion were detailed in tabular format [7] (Figure 1).

### 2.5. Data Selection and Extraction

The extracted data consisted of: 1. General information; 2. Study identification (authors, title, year published); 3. Study characteristics (setting, objectives, study design, sample size, inclusion and exclusion criteria); recruitment methodology—e.g., retrospective or prospective cohort—controls, follow-up length; 4. Participants’ characteristics (age, morbidities; reason for testicular imaging study); 5. Variables that could influence outcomes (age, lesion size, lesion ultrasound characteristics, symptoms, tumor markers, hormonal status, infertility, history of cryptorchidism, history of testicular tumor); 6. Outcomes (malignant or benign histology); 7. Effect size for associations reported between the identified variables and outcomes.

Only the information that was relevant to this systematic review research question was extracted. If the same data were reported in multiple study publications, the duplicates were deleted to minimize the overrating of any variable or outcome investigated in the same sample.

## 3. Results

The initial main literature search provided 2126 articles (37 from Embase, 743 from PubMed, 640 from Web of Science Core Collection, 606 from Cochrane Central Register, 80 from Clinical trials and 20 from MedRxiv) (Figure 1). Following screening of titles and abstracts and removing duplicates, we retrieved 112 full-text articles. Fifty-five articles were excluded after full text review. Ultimately, 57 studies were included in the final list. Thirty-nine of them described cases of impalpable testicular masses diagnosed incidentally by US [8,9,10,11,12,13,14,15,16,17,18,19,20,21,22,23,24,25,26,27,28,29,30,31,32,33,34,35,36,37,38,39,40,41,42,43,44,45,46], and 47 included information about the histology of small testicular masses that underwent surgical treatment [5,6,8,9,10,11,12,13,14,15,16,17,24,25,26,28,29,30,31,32,33,34,35,36,39,40,42,43,44,46,47,48,49,50,51,52,53,54,55,56,57,58,59,60,61,62]. The characteristics of the included studies are reported in tabular format (Supplementary Data—Supplementary Table S1).

### 3.1. Small Nonpalpable Testicular Masses Detected by Ultrasound (US)

Several retrospective studies report series of US examinations, both for the study of infertility (10 out of 23, 43%) and in the general male population consulted for various indications (e.g., trauma, orchialgia, palpable testicular mass, swelling, varicocele, hydrocele or other scrotal lesions) (13 out of 23, 57%). Although six of these studies (26%) did not specify the total number of US performed in the study period, in total, the experience of over 31,899 ultrasounds is summarized (Table 1).

The percentage of cases where testicular masses were diagnosed, for all patients who underwent US investigation, ranged from 0.2% on a large series of 5104 US examinations [9] to 3.4% on a large clinical trial [30], and was up to 6% [28] and 34% [23] in smaller, less representative studies. On average, testicular masses were detected in 1.74% of patients undergoing US examination across all series.

The proportion of patients diagnosed with STMs among those consulted for infertility ranged from 0.8% [41] to 3% [11], but was up to 6% [28] and 34% [23] in smaller studies reporting less than 200 US. The average percentage of patients screened for infertility and in whom a testicular mass was diagnosed was 2.86%. On the other hand, patients who underwent US for various indications had a percentage of diagnosed masses ranging from 0.2% [9] to 3.4% [30] in large studies reporting over 5000 cases (and in smaller case series up to 8% [44]). The average incidence of STMs in this setting was 1.41%. Overall, the incidence of STMs amongst men consulted for infertility (2.86%) appears to be higher than for men who underwent US for various indications (1.41%).

We defined incidental masses as nonpalpable lesions diagnosed only on US evaluation with less than 1 cm. In this context, we observed that a substantial number of studies (12 out of 23, 52%) selected and reported only incidental testicular masses in their case series (100%) [9,11,14,19,20,21,27,28,29,37,39,46]. Moreover, analyzing studies that report both palpable and nonpalpable masses, the proportion of incidental masses ranged from 37% [15] to 87.5% [38] (100% in a smaller sample reported by Sakamoto et al. [41]). Based on this data, the average percentage of nonpalpable masses across these seven studies [15,16,18,30,38,41,45] was 59% of all diagnosed masses. Additionally, we observed that case series reporting bigger mass size ranges often resulted in a lower percentage of impalpable tumors [15,16].

Importantly, the final histology of small testicular masses diagnosed by US was likely to be benign. From the five studies [9,18,21,27,45] that reported a higher rate of malignant tumors on final histology (>50%), Comiter et al. reported the highest (87% malignancies) [18]. Additionally, five articles reported a 50/50 distribution of malignant and benign lesions based, however, on small case series [24,28,37,39,44]. The remaining 11 studies reported predominantly benign lesions, comprising 100% [14,20], 93% [23], 88% [46], 87.5% [38], 78% [29], 75% [16,33], 67% [11], 52% [15] and 51% [30] of diagnosed testicular masses. Analyzing all data together, the average percentage of benign tumors was 58.31% (versus 41.69% malignant) in this context. The trend for predominance of benign lesions is slightly more noticeable in studies reporting only incidental lesions [9,11,14,19,20,21,27,28,29,37,39,46], with an average percentage of 63.24% of these selected tumors having benign histology.

### 3.2. Nonpalpable Small Testicular Masses in Surgical Case Series

Impalpable and pre-operatively undiagnosed testicular masses were also reported in surgical case series retrieved in our search (16 articles) (Table 2). Six of these works selected and surgically treated only incidental STMs (100%) [13,26,35,36,40,42]. In the remaining case series, which included both palpable and nonpalpable lesions, we can observe that incidental masses accounted from 20% [8] to 83% [17] of all enucleated testicular lesions, with an average percentage of 46.5% impalpable masses.

It must be noted that in a crushing majority of these case series, STMs were more often benign than malignant, i.e., over 50% benign lesions (14 out of 16, 87.5%). Benign lesions ranged from 64% of the total excised masses in a considerably sized study by Bojanic and colleagues [12] to over 90% in several other publications [8,10,22]. In total, final histology was reported for 283 surgically treated masses, with 192 being benign and 91 revealed to be malignant. Overall, the average percentage of benign lesions enucleated across these studies is 67.84%.

### 3.3. Frozen Section Examination

Then, we looked into studies describing data that allow us to evaluate the accuracy of frozen section in properly identifying STM histology. We stated the accuracy of FSE as the sensitivity for the detection of malignancy. We analyzed all masses (*n* = 1931) that were treated surgically (either by partial or radical orchiectomy) (Table 3). Out of all small masses undergoing surgery, 794 were benign on final pathology report (41.12%) and 1137 were malignant (58.88%).

Our results showed that FSE is highly reliable for detecting malignant lesions throughout the reported case series, reaching 100% accuracy in 25 out of 38 studies (66%) [8,10,12,13,14,15,16,17,22,25,26,29,31,34,35,40,42,43,46,48,51,53,55,57,58]. Good accuracy was also reported in five studies, namely, by Connolly and colleagues (96.1%) [49], in the 14 years of experience reported by Silverio et al. (96%) [56], by Matei et al. (93%) [54], in an earlier study by Dell’Atti (84.3%) [50] and by Ferretti et al. (83.33%) [52].

Lower accuracy (defined by us as <80%), was reported in 8 out of 38 case series. Bieniek and colleagues reported 78.60% accuracy of FSE for the detection of malignancy [11], Muller et al. correctly diagnosed 75% of malignant lesions by FSE [36], 66.7% accuracy was found in the report by Fabiani et al. [24], 63.33% accuracy was described by Ayati et al. [47], and 62.5% sensitivity for detection of malignancy was reported by Avci and colleagues [9]. The last three of these reported under 50% accuracy for FSE [28,33,39]. Overall, the average accuracy of FSE amongst all studies was 93.05%.

In these surgical series, STMs appear to be more frequently benign if smaller in size. In fact, for studies reporting a mean tumor size under 2.5 cm (29 articles), the average rate of benign lesions was 55.77%. For lesions under 1 cm mean diameter (15 articles), the average percentage of benign masses was 68.78% (versus 31.22% malignant).

Analyzing reported patient ages, we verified that testicular tumors were more frequent in young patients, with every included study displaying a mean patient age comprised within the third to fifth decade of life. In articles describing mean age of their sample between 25 and 34.9 years (*n* = 14), the average percentage of benign lesions was 52.01%. For a cut-off defined by reported mean ages of 35 to 39.9 years (17 studies) we found 47.10% benign lesions. For the seven studies reporting mean patient age equal to or over 40 years, the average rate of benign tumors was 43.41%. Despite the interesting results, there was not a clear correlation between patient age and malignant small testicular masses.

At last, we highlight that TSS coupled with FSE allowed for the sparing of an ample percentage of testicles. TSS is described in 41 out of the 47 analyzed studies and allowed for organ-sparing procedures in 673 patients. The average percentage of lesions treated by TSS across these series was 34.9%.

### 3.4. Predictive Factors for Malignancy of Testicular Masses

The most difficult aspect in clinical practice is to decide between organ-sparing surgery or radical orchiectomy after the diagnosis of a small incidental testicular mass. Only a few studies reported an analysis on preoperative predictive variables.

Our gathered results showed that tumor size is the most frequently analyzed variable for predicting malignancy. In studies reporting mean size of the testicular masses under 2.5 cm, these lesions were more often benign (55.77%) than malignant; with an increased percentage of benign lesions for masses under 1 cm (68.78%) (Table 3). Smaller mass size was consistently associated with benign histology within the five studies that specifically analyzed this variable [6,30,54,60,61]. Some of these articles established cut-offs to predict malignancy, reporting that testicular lesions with diameters <2 cm (*p* < 0.001) [54], <18.5 mm (87% sensitivity and 83% specificity; *p* < 0.05) [6], <5 mm (*p* = 0.002) [61] and <4.5 mm (sensitivity = 0.87; specificity = 0.64) [60] are correlated with benign histology. The identifiable trend is that smaller lesions are more often benign.

We found that patient age was not a predictive factor for malignancy. For articles reporting at least or over 80% benign lesions (10 out of 38 articles reporting mean age or sufficient data to calculate it; 26.3%), the average mean age of the patient population was 36.55 years. Conversely, for studies where 80% or more masses were malignant (2 out of 38; 5.3%), the average mean age was 38.65 years. The average percentage of benign lesions was 52.01% across articles reporting mean ages between 25 and 34.9 years, 47.10% for ages between 35 and 39.9 years and 43.41% for a mean patient age equal to or over 40 years (Table 3). This was exemplified in the clinical trial by Isidori and colleagues, who also found no significant difference between ages in malignant and benign tumor groups (*p* = 0.927) [30]. However, data in an 81 patient case series indicated that malignant lesions were associated with younger individuals (mean age for benign histology was 43.6 years, and for malignant was 32.6 years; *p* = 0.005) [61].

**Figure 2:**
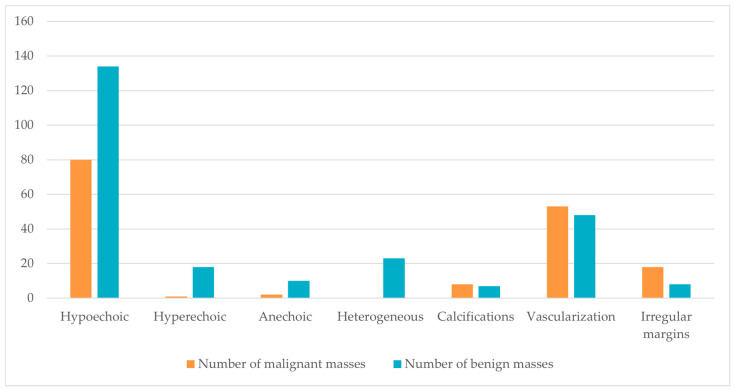
Final histology of small testicular masses according to ultrasonographic characteristics.

Subsequently, we evaluated whether certain aspects of the lesions on US may help to predict the final histology. For that, we used the data regarding ultrasonographic characteristics available on Table 1 and Table 2. From a total of 314 masses (from which we have US data), we observed that hypoechoic focal areas were the most common findings, with 214 testicular masses being described as such across all case series. Both benign and malignant lesions frequently presented as hypoechoic on scrotal US-134 benign lesions (62.6%), 80 malignant lesions (37.4%). Nineteen hyperechoic lesions were described, of which only 1 was malignant (5.3%). In its turn, anechoic lesions were almost always benign (10 out of 12, 83.3%) and cystic in nature. Calcifications are rare on both benign and malignant lesions, with only 15 calcified lesions described—8 malignant (53.3%) and 7 benign (46.7%). Internal vascularization on color Doppler US was found in 101 masses across the analyzed case series and was frequent in both benign (47.5%) and malignant lesions (52.5%). Finally, irregular margins were more common in malignant tumors (18 out of 26, 69.2%). These findings are summarized in graphical form (Figure 2). In general, malignant lesions seem to have irregular margins; whereas benign lesions might be hypoechoic, hyperechoic, or anechoic, but are more likely to display regular margins and present as hypoechoic. Interestingly, malignant lesions were not heterogeneous (23 masses, 100% benign).

## 4. Discussion

In this study we verified that testicular masses are relatively infrequent, affecting 1.74% of men undergoing scrotal US examination, and are usually benign on final histology. TSS coupled with frozen section examination is a valid option for the management of STMs due to the high reliability of FSE. We observed that lesion size and ultrasonographic characteristics may help to predict the likelihood of malignancy and might be a useful tool for conservative management.

The nonpalpable small testis masses represent a management dilemma for the urologist, who must balance the risk of malignancy with the iatrogenic results of removing testicles that might bear benign lesions. Most patients with small testicular masses do not have a clear presentation, and highly disparate clinical patterns are described in the literature. Available tools for the clinician are symptomatic enquiries, epidemiological risk factors, serum tumor markers and scrotal US. However, in most cases the definitive diagnosis of the small lesions incidentally discovered cannot be established.

An important question that remains to be answered is how frequently small testicular masses are benign or malignant. We verified that on US series, STMs are most often benign—58.31% (versus 41.69% malignant) and across surgical case series comprise 41.12% (versus 58.88% malignant) of masses removed.

The trend for predominance of benign lesions was more noticeable in studies reporting only incidental lesions [9,11,14,19,20,21,27,28,29,37,39,46], with an average percentage of 63.24% of these selected tumors having benign histology. Clinically, these testicular lesions were innocent, being nonpalpable in over 59% of the cases (Table 1 and Table 2) [15,16,18,30,38,41,45]. Interestingly, within surgically treated small testicular masses (Table 3), the percentage of benign lesions seemed to be lower (41.12%) compared to US series. This can be partially explained by the fact that some testicular masses amongst these case series were followed by US without surgical removal. These lesions remained with stable size and characteristics over long follow up periods, consistent with an ultimately benign nature [30,60]. As such, we can conclude that when considering STMs (<2.5 cm), we may be more likely to be dealing with a benign testicular lesion.

We also verified that the incidence of STMs on US among men consulted for infertility (2.86%) appeared to be significantly higher than in men examined for various indications (1.41%) (1.74% across all studies). However, several of the latter included also poorly reported numbers of patients consulted for infertility, which complicates the analysis. The apparently higher incidence of testicular tumors in the infertile population might be justified by an increased screening and related pathologies identified as risk factors for testicular cancer, such as cryptorchidism, Klinefelter syndrome or gonadal dysgenesis syndrome [33]. While infertility is regarded as a risk factor for TC, we verified that the average proportion of benign tumors amongst men consulted for infertility was higher (74.79%) than for men who underwent US for various indications (59.87%). This might be partially explained by the smaller sample of studies reporting only infertile populations (11 out of 38 articles, totaling 119 cases) or by the fact that infertile men are submitted to more US screening than the general population (detecting benign masses that would otherwise never manifest themselves). Thus, even though STMs might be more frequent in populations of infertile men, and infertility is a risk factor for testicular cancer, these lesions are still likely to be benign.

An utmost relevant and debatable question is the appropriate management for STMs. Oncologic outcomes of TSS and radical orchiectomy after inguinal exploration and FSE for patients both with benign and malignant final pathology are similar [8,22,25,36,43]. Although similarly good oncologic results and favorable functional outcomes were reported in many series, there is no clear consensus on which patients partial orchiectomy is to be applied. Amongst the case series we analyzed, the most widely accepted indications for considering TSS were a nonpalpable testicular mass diagnosed incidentally by US examination [9,13,22,24,28,30,36,39,40,42,47], lesions under the size of 25 mm [8,25]; testicular lesion volume <30% of the whole testis, not clearly suggestive of malignancy, with negative tumor markers and without disseminated disease [8,34,43]. However, TSS risks should be considered and include disruption of the predictable lymphatic spread pattern, positive surgical margins and unrecognized lesions or carcinoma in situ remaining in the preserved testis [47]. Recurrence can be explained by the presence of multifocality and/or testicular intraepithelial neoplasia, which is almost invariably present in testicular parenchyma adjacent to a germ cell tumor [12]. In order to plan a targeted intraoperative screening of GCNIS by FSE, it is useful to know that although GCNIS foci may be present very close to the STM or as skip lesions in the surrounding parenchyma, there may be a linear correlation between the size of STMs and the distance of GCNIS foci to the mass [63]. Disease recurrence should be managed resorting to symptomatic enquiries, scrotal physical examination, tumor marker assays and ultrasonographic evaluations.

TSS is currently not advisable without intraoperative frozen section examination (FSE). This makes intraoperative histopathological diagnosis possible, guiding how the treatment is to be completed. During surgery, the lesion can be identified either with palpation or with intraoperative US, especially useful in case of smaller impalpable masses [13,14,24,28,50]. The standard form of treatment after the detection of malignancy by FSE is conversion of the procedure to radical orchiectomy, since there is a potentially high local recurrence rate in these patients. Conversely, benign FSE results sustain the option for conservation of the remaining healthy testis. The limitations of FSE should always be taken into account and in doubtful cases the clinician should consider every available evidence from clinical data, laboratory, radiology, and pathology to decide whether or not to proceed to radical orchiectomy.

We found that FSE is consistent and provides up to 100% sensitivity for the detection of malignancy (average 93.05% across all studies). We verified that TSS coupled with FSE aided in preventing unnecessary radical orchiectomy in a high percentage of patients, representing 673 (34.9%) testicles spared. These are encouraging numbers but fall short of the percentage of enucleated tumors that are actually benign (41.12%). This can be explained by the exclusive adoption of RO in some studies or by inaccurate or inconclusive FSEs hindering the use of TSS for some ultimately benign lesions. These results clearly support the use of FSE to decide on a conservative or radical surgical approach for STMs. All patients must be aware that they may need a radical orchidectomy if frozen section assessment is positive for cancer or deemed inconclusive or inaccurate [31]. Therefore, close collaboration between the pathologist and the urologic surgeon is required when testis sparing surgery is contemplated [42]. All things considered, TSS paired with FSE is a reliable option for the management of STMs and is crucial to preclude the removal of testicles bearing benign lesions.

Mass diameter was studied as a surrogate marker for malignancy. As surgical case series guarantee the most reliable data regarding final histology of testicular masses, we analyzed the data available on Table 3 and found that for studies reporting a mean tumor size within their cases of under 2.5 cm, the mean rate of benign lesions was 55.77%. Interestingly, for under 1 cm mean diameter masses, an average 68.78% were benign (versus 31.22% malignant). This warrants that usually (>50%), small testicular masses are benign and, therefore, might be managed conservatively. The proportion of benign lesions in smaller masses was high and there was a direct correlation between the increasing size and the rate of malignant lesions. Different studies looked at mass dimensions to identify for which size it would be safe to perform TSS or even follow these lesions with US (Table 3) [6,54,60,61]. We conclude that it is still debatable exactly how small these testicular masses should be to justify the option for TSS, but the most consensual maximum acceptable size gravitates around 1 cm (considering the higher percentage of benign lesions within this size range). We believe that this range is widely accepted not only because larger tumors are at higher risk for malignancy but also because preserving sufficient functioning parenchyma may be difficult after enucleation of a lesion exceeding this size [22].

Ideally, US features would be of interest to distinguish benign from malignant masses. Although the available reports assume inconsistent shapes when describing the ultrasonographic characteristics of their cases, in some studies it was possible to associate benign or malignant final histology with the presentation of the mass on diagnostic US. The most frequent finding is a hypoechoic lesion, which can be frequently benign (62.6%) or malignant (37.4%). Calcifications and vascularization are characteristics of both benign and malignant masses. In general, malignant lesions seem to have irregular margins. On the other hand, benign lesions might be hypoechoic, hyperechoic or anechoic (although a malignant teratoma can mimic this cystic appearance [22,29]) but are more likely to display regular margins and appear as hypoechoic. Interestingly, malignant lesions were not heterogeneous, but this conclusion is based on a small sample.

We might say that although the imaging features of benign solid testicular lesions vary extensively, and the available data are contradictory at some points, a benign testicular tumor can be suspected on US for a small testicular mass (less than 1 cm) with regular margins, frequently hypo or anechoic. In fact, based on the premise that STMs are frequently benign, and due to high probability of benignity, some selected testicular masses within the reported case series were managed only by serial US examinations, and most exhibited no significant growth during prolonged follow up [10,11,19,20,41,42]. US surveillance is increasingly considered as an alternative to prevent unnecessary surgical intervention for very small testicular masses [19,23,46,60].

As to increase the accuracy of non-invasive characterization of STMs, experience with imaging tools other than conventional US is necessary. Nowadays, contrast-enhanced ultrasound (CEUS) is a promising diagnostic exam for testicular masses, offering a great diagnostic performance with an accuracy of 0.96 in detecting malignant masses, as reported in a recent meta-analysis [64].

However, further research in the field of preoperative predictors of malignancy in STMs is still required. It would be useful for the clinician to be able to rely on more imaging tools or novel doseable blood markers for malignant disease, such as blood-based miRNA, in order to select patients eligible for conservative treatment.

Our study has some limitations. The first is the retrospective nature of the available studies, comprised mainly of case series and exposure to their potential patient selection and report biases. The number of patients included in each individual study group was limited, given the relative rarity of STMs. Additionally, the definitions of incidental and small testicular masses are disparate in the literature. Incidental masses are inconsistently reported either as an impalpable testicular mass diagnosed on ultrasound for the study of infertility (or symptoms such as testicular pain) or only as a mass diagnosed in the absence of any symptoms or during physical examination for unrelated nonurological complaints. In its turn, STMs are defined under variable cut-offs of 5 mm, 10 mm, 15 mm, 20 mm, 25 mm or even 5 cm. Almost no study reported effect size associations between analyzed variables, and thus, the strength of the evidence available is limited.

## 5. Conclusions

Small testicular masses are commonly diagnosed due to the widespread use of scrotal ultrasound evaluation. These testicular lesions are often benign, especially if impalpable and/or sub-centimetric. Our study concludes that FSE is an accurate tool to discriminate between benign and malignant neoplastic lesions, supporting the use of TSS. Clinical and US patterns are not reliable as parameters for surveillance protocols without FSE, but available data endorse that benign lesions are usually smaller than <1 cm, have regular margins and are often hypoechoic in appearance. Future research with new imaging tools or novel biomarkers might support clinical management.

## Figures and Tables

**Table 1:** Prevalence of nonpalpable testicular masses detected on US for several indications.

Authors (Year)	Period	Ultrasound Examinations (N)	Indications for US	Mass Size US (mm)	Ultrasonographic Characteristics	Diagnosed Masses N (%)	Nonpalpable N (%)	Malignant N (%)	Benign N (%)	No Histology N	References
Avci et al. (2008)	2002–2007	5104	Various indications	mean 6	All hypoechoic.	11 (0.2%)	11 (100%)	5 (56%)	4 (44%)	2	[9]
Bieniek et al. (2017)	2001–2014	4088	Infertility	mean 4.14	All malignant lesions demonstrated vascularity.	120 (3%)	120 (100%)	6 (33%)	12 (67%)	102 *	[11]
Buckspan et al. (1989)	NA	~400	Infertility	range (3–6)	ND	4 (1%)	4 (100%)	0 (0%)	4 (100%)	0	[14]
Carmignani et al. (2004)	2000–2003	560	Infertility	range (4–26)	1 lesion showed hypervascularization, revealed to be a diffuse Leydig Cell hyperplasia.	8 (1.4%)	4 (50%)	2 (25%)	6 (75%)	0	[16]
Carmignani et al. (2003)	2000–2002	1320	Various indications	range (3–24)	ND	27 (2%)	10 (37%)	13 (48%)	14 (52%)	0	[15]
Comiter et al. (1995)	1985–1994	3019	Various indications	mean 11.6	12 hypoechoic (11 malignant, 1 benign); 1 homogeneously echogenic (benign); 2 calcified (regressed malignant tumor).	15 (0.5%)	8 (53%)	13 (87%)	2 (13%)	0	[18]
Connolly et al. (2006)	1997–2004	1544	Various indications	mean 4.9	8 hypoechoic (7 benign, 1 malignant); 3 anechoic (benign); 1 hyperechoic (benign).	12 (0.8%)	12 (100%)	1	0	11 *	[19]
Corrie et al. (1991)	NA	NA	Various indications	mean 14.8	Two masses resolved on US follow-up. 1 hypoechoic and 1 hyperechoic at presentation.	5	5 (100%)	0	3 (100%)	2 *	[20]
Csapo et al. (1988)	NA	NA	Various indications	NA	All hypoechoic.	2	2	2	0	0	[21]
Eifler et al. (2008)	1995–2006	145	Infertility	range (<5; >10)	10 hyperechoic (benign); 19 heterogeneous (benign); 18 hypoechoic (1 malignant, 17 benign). 5 Hypervascular (4 benign, 1 malignant).	49 (34%)	NA	1 (7%)	13 (93%)	35	[23]
Fabiani et al. (2014)	a 43-month period	717	Various indications	<10	ND	8 (1.1%)	NA	4 (50%)	4 (50%)	0	[24]
Hindley et al. (2003)	2000–2001	NA	Various indications	range (4–25)	All hypoechoic.	4	4 (100%)	3 (75%)	1 (25%)	0	[27]
Hopps and Goldstein (2002)	1995–2001	65	Infertility	mean 7.6	All hypoechoic.	4 (6%)	4 (100%)	2 (50%)	2 (50%)	0	[28]
Horstman et al. (1994)	1984–1992	1600	Various indications	mean 8.8	7 hypoechoic (6 benign, 1 malignant); 1 hyperechoic (benign); 1 cystic/anechoic (malignant).	9 (0.6%)	9 (100%)	2 (22%)	7 (78%)	0	[29]
Isidori et al. (2014)	2006–2012	5720	Various indications	mean 7	Hypoechoic (39 malignant, 34 benign; *p* = 0.320); Internal vascularization (28 benign, 42 malignant; *p* < 0.001); Intratumorous calcifications (4 benign, 6 malignant; *p* = 0.010); Irregular margins (8 benign, 18 malignant; *p* = 0.039).	197 (3.4%)	115 (58%)	44 (49%)	46 (51%)	25	[30]
Lagabrielle et al. (2018)	1989–2008	NA	Infertility	median 8.5	ND	32	NA	8 (25%)	24 (75%)	0	[33]
Onur et al. (2008)	NA	NA	Infertility	7.15	All hypoechoic.	2	2 (100%)	1 (50%)	1 (50%)	0	[37]
Pierik et al. (1999)	NA	1372	Infertility	mean 14	NA	16 (1.2%)	14 (87.5%)	2 (12.5%)	14 (87.5%)	0	[38]
Powell and Tarter (2006)	a 36-month period	1040	Various indications	mean 5.5	All hypoechoic.	4 (0.4%)	4 (100%)	2 (50%)	2 (50%)	0	[39]
Sakamoto et al. (2006)	1998–2004	545	Infertility	NA	ND	4 (0.8%)	4 (100%)	NA	NA	4 *	[41]
Tackett et al. (1986)	1980–1984	249	Various indications	NA	ND	20 (8%)	NA	10 (50%)	10 (50%)	0	[44]
Tal et al. (2004)	1992–2002	NA	Infertility	median 13	NA	11	8 (73%)	6 (67%)	3 (33%)	2	[45]
Toren et al. (2010)	2001–2008	4418	Various indications	mean 4.3	All hypoechoic.	46 (1%)	46 (100%)	1 (12%)	7 (88%)	38	[46]

US = ultrasound; N = number; NA = not available; ND = no sufficient data to associate reported echographic patterns with benign or malignant final histologies. * With benign behavior (no growth, slow growth or complete resolution) on prolonged US follow-up.

**Table 2:** Prevalence of nonpalpable testicular masses in surgical case series.

Authors (Year)	Period	Indications for US	Mass Size US (mm)	Ultrasonographic Characteristics	Diagnosed Masses N	Nonpalpable N (%)	Malignant N (%)	Benign N (%)	No Histology N	References
Ates et al. (2016)	2010–2014	Various indications	mean 16	Hypoechoic (12 benign); Calcifications (3 benign).	15	3 (20%)	1 (7%)	14 (93%)	0	[8]
Benelli et al. (2017)	2005–2014	Organ-sparing surgery	mean 13.6	10 hypothesized benign on US: 7 hypoechoic/avascular (3 necrosis, 4 underwent only US surveillance), 2 anechoic/avascular (2 epidermoid cysts), 1 heterogeneous/avascular (sertolli cell tumor); 4 not settled: 3 heterogeneous/avascular (1 necrosis, 2 epidermoid cyst), 1 hypoechoic/avascular (necrosis); 4 hypothesized malignant: hyperechoic/hypervascularized (3 LCT, 1 seminoma).	18	9 (50%)	1 (7%)	13 (93%)	4	[10]
Bojanic et al. (2017)	NA	Various indications	mean 11.4	NA	28	18 (64%)	10 (36%)	18 (64%)	0	[12]
Browne et al. (2003)	NA	Various indications	NA	All hypoechoic.	3	3 (100%)	2 (67%)	1 (33%)	0	[13]
Colpi et al. (2005)	2001–2004	Infertility	mean 4.33	4 hypoechoic (3 benign, 1 malignant); 2 anechoic (benign).	6	5 (83%)	1 (17%)	5 (83%)	0	[17]
De Stefani et al. (2012)	2004–2011	Various indications	mean 14.3	2 Hypoechoic (1 malignant, 1 benign); 2 Hypervascular (benign); 4 Cystic/anechoic (1 malignant, 3 benign).	23	18 (78%)	2 (9%)	21 (91%)	0	[22]
Gentile et al. (2013)	2009–2013	Various indications	mean 9.5	Hypoechoic lesion with vascularization for Leydig cell tumors (5 cases); 1 Hyperechoic lesion (adenomatoid tumor); 1 Hypoechoic lesion (fibromyxoid liposarcoma); 1 Irregular with a focal hypoechoic lesion without vascularization (seminoma).	15	10 (67%)	2 (13%)	13 (87%)	0	[25]
Hallak et al. (2009)	NA	Infertility	mean 6.7	All hypoechoic and vascularized.	6	6 (100%)	1 (17%)	5 (83%)	0	[26]
Khan et al. (2018)	2013–2017	Various indications	Mean 9.8	NA	12	3 (25%)	3 (25%)	9 (75%)	0	[31]
Kizilay et al. (2019)	2000–2017	Organ-sparing surgery	mean 11	ND	27	18 (67%)	9 (33%)	18 (67%)	0	[32]
Leonhartsberger et al. (2014)	2003–2010	Organ-sparing surgery	mean 14.8	NA	68	18 (27%)	43 (63%)	25 (37%)	0	[34]
Leroy et al. (2003)	1996–2002	Various indications	mean 7.5	NA	15	15 (100%)	4 (27%)	11 (73%)	0	[35]
Muller et al. (2006)	2000–2005	Various indications.	mean 3.5	17 hypoechoic (14 benign, 3 malignant); 2 vascularized (malignant).	20	20 (100%)	4 (20%)	16 (80%)	0	[36]
Rolle et al. (2006)	2003–2005	Various indications	mean 5.7	All hypoechoic.	7	7 (100%)	1 (14%)	6 (86%)	0	[40]
Sheynkin et al. (2004)	1998–2002	Various indications	NA	ND	9	9 (100%)	2 (25%)	6 (75%)	1 *	[42]
Shilo et al. (2012)	(last 15 years)	Various indications	mean 16.4	NA	16	4 (25%)	5 (31%)	11 (69%)	0	[43]

US = ultrasound; N = number; NA = not available; ND = no sufficient data to associate reported echographic patterns with benign or malignant final histologies. * With benign behavior (no growth, slow growth or complete resolution) on prolonged US follow-up.

**Table 3:** Histology of STMs and accuracy of FSE.

Authors (Year)	Number of Cases (N)	Mean Age (Years)	Tumor Size Mean (Range) mm	Definitive Histology-N (%)		Accuracy of FSE	Number of TSS (%)	Inclusion Criteria for Explorative Surgery with FSE	References
				Malignant	Benign				
Ates et al. (2016)	15	25.33	16 (5–26)	1 (7%)	14 (93%)	100%	14 (93%)	Lesion size <25 mm and testicular lesion volume <30% of the whole testis.	[8]
Avci et al. (2008)	11	median 24	6 (4–9)	5 (56%)	4 (44%)	62.50%	0	Nonpalpable testicular masses discovered by US.	[9]
Ayati et al. (2014)	10	32.2	10.6 (6–19)	6 (60%)	4 (40%)	63.33%	4 (40%)	Nonpalpable testicular masses discovered by US.	[47]
Benelli et al. (2017)	18	33.3	16.8	1 (7%)	13 (93%)	100%	14 (100%)	NA	[10]
Bieniek et al. (2017)	120	36.7	4.14	6 (33%)	12 (67%)	78.60%	13 (72%)	Subcentimeter testicular mass. (<10 mm)	[11]
Bojanic et al. (2017)	28	35.3	11.4 (5–20)	10 (36%)	18 (64%)	100%	26 (93%)	Testicular lesions <20 mm and no evidence of metastatic disease.	[12]
Bozzini et al. (2014)	86	38	24 (4.4–100)	40 (47%)	39 (45%)	100%	32 (37%)	NA	[48]
Browne et al. (2003)	3	37	NA	2 (67%)	1 (33%)	100%	1 (33%)	Nonpalpable testicular masses discovered by US.	[13]
Buckspan et al. (1989)	4	range (23–40)	(3–6)	0 (0%)	4 (100%)	100%	4 (100%)	NA	[14]
Carmignani et al. (2004)	8	37.3	(4–26)	2 (25%)	6 (75%)	100%	4 (50%)	Lesions with clear-cut ultrasonographic edges and no history of recent genital infections.	[16]
Carmignani et al. (2003)	27	41.2	(3–24)	13 (48%)	14 (52%)	100%	15 (56%)	NA	[15]
Colpi et al. (2005)	6	39.8	(3–6)	1 (17%)	5 (83%)	100%	5 (83%)	NA	[17]
Connolly et al. (2006)	80	35	25 (5–50)	52 (65%)	28 (35%)	96.1%	25 (31%)	NA	[49]
De Stefani et al. (2012)	23	30.6	16.5	2 (9%)	21 (91%)	100%	21 (91%)	Nonpalpable or small testicular masses (<2 cm) not clearly suggestive of malignancy and without disseminated metastasis.	[22]
Dell’Atti (2016)	49	33	12.3 (5–15)	35 (71%)	14 (29%)	84.3%	49 (100%)	Size of the mass <1.5 cm.	[50]
Dell’Atti et al. (2018)	77	36.5	median 13.4 (5–20)	49 (64%)	28 (36%)	100%	37 (48%)	Masses under 1.5 cm.	[51]
Fabiani et al. (2014)	8	31.75	5 (2.5–8)	4 (50%)	4 (50%)	66.7%	3 (38%)	Small (<1 cm) incidental nodules.	[24]
Ferretti et al. (2014)	25	31.9	11.66	20 (80%)	5 (20%)	83.33%	19 (76%)	Bilateral synchronous tumor, and tumor in a single testicle.	[52]
Galosi et al. (2016)	28	38	9.3 (2.5–15)	6 (21%)	22 (79%)	100%	17 (61%)	A single testis lesion measuring less than 15 mm at ultrasound.	[53]
Gentile et al. (2013)	15	44.3	10.5	2 (13%)	13 (87%)	100%	13 (87%)	Diameter <25 mm.	[25]
Haas et al. (1986)	233	NA	NA	161 (69%)	72 (31%)	NA	21 (29%)	Inguinal explorations performed for the suspicion of cancer.	[5]
Hallak et al. (2009)	6	35.8	6.7	1 (17%)	5 (83%)	100%	6 (100%)	NA	[26]
Hopps and Goldstein (2002)	4	NA	7.6	2 (50%)	2 (50%)	0%	2 (50%)	Nonpalpable testicular masses discovered by US.	[28]
Horstman et al. (1994)	9	35.88	8.8 (3–15)	2 (22%)	7 (78%)	100%	NA	NA	[29]
Isidori et al. (2014)	115 *	34	median diameter malignant: 12; benign: 6 (*p* < 0.001)	44 (49%)	46 (51%)	NA	47 (52%)	Nonpalpable lesions <1.5 cm.	[30]
Khan et al. (2018)	12	40	9.8 (3–18)	3 (25%)	9 (75%)	100%	9 (75%)	NA	[31]
Kizilay et al. (2019)	27	29.7	11 (2–18)	9 (33%)	18 (67%)	NA	27 (100%)	NA	[32]
Lagabrielle et al. (2018)	32	36	8.5	8 (25%)	24 (75%)	43%	32 (100%)	Incidental testis tumors treated by partial orchiectomy in a population of infertile men.	[33]
Leonhartsberger et al. (2014)	68	38.9	14.8 (2–30)	43 (63%)	25 (37%)	100%	33 (49%)	Marker-negative clinical stage I testicular tumors <30 mm and marker-positive tumors in case of a tumor in a singular testis.	[34]
Leroy et al. (2003)	15	34.3	7.5 (4–16)	4 (27%)	11 (73%)	100%	9 (60%)	NA	[35]
Li et al. (2017)	101 *	median 42	4.4 (1–10) benign <4.5 mm (*p* < 0.05)	15 (60%)	10 (40%)	NA	3 (12%)	NA	[60]
Matei et al. (2017)	144	34	15 benign <20 mm (*p* < 0.001)	80 (56%)	64 (44%)	93%	57 (40%)	Masses < 1 cm, nonpalpable, multiple or with unusual presentation.	[54]
Muller et al. (2006)	20	36.4	3.5 (1.5–5.0)	4 (20%)	16 (80%)	75%	16 (80%)	Incidental intratesticular masses of ≤5 mm in diameter.	[36]
Passarella et al. (2003)	11	43	NA	2 (18%)	9 (82%)	100%	7 (64%)	Masses suspected to be benign.	[55]
Powell and Tarter (2006)	4	26.75	5.5 (5–6)	2 (50%)	2 (50%)	0%	2 (50%)	Nonpalpable testicular masses discovered by US.	[39]
Rolle et al. (2006)	7	42	5.7 (2.5–16)	1 (14%)	6 (86%)	100%	6 (86%)	Nonpalpable hypoechoic testicular lesions.	[40]
Scandura et al. (2018)	81	40 malignant: 32.6; benign: 43.6 (*p* = 0.005)	range (1.7–9.6) benign <5 mm (*p* = 0.002)	25 (31%)	56 (69%)	NA	4 (5%)	NA	[61]
Sheynkin et al. (2004)	9	34	NA	2 (25%)	6 (75%)	100%	1 (11%)	Nonpalpable testicular masses discovered by US.	[42]
Shilo et al. (2012)	16	32.38	16.44 (8–25)	5 (31%)	11 (69%)	100%	11 (69%)	Well-defined small (<2.5 cm) testicular lesions and no serum marker elevation and no evidence of metastasis.	[43]
Shilo et al. (2012)	127	NA	ranges (<10; >20) malignant: mean 41 benign: mean 15 (*p* < 0.05)	120 (94%)	7 (6%)	NA	NA	NA	[6]
Shtricker et al. (2015)	85	NA	NA	71 (84%)	14(16%)	NA	NA	NA	[62]
Silverio et al. (2015)	159	36	35 (5–120)	107 (67%)	52 (33%)	96%	32 (20%)	NA	[56]
Tackett et al. (1986)	20	NA	NA	10 (50%)	10 (50%)	NA	3 (15%)	Suspicion of testicular neoplasm.	[44]
Tokuc et al. (1992)	26	NA	NA	24 (92%)	2 (8%)	100%	0	NA	[57]
Toren et al. (2010)	41	35	4.3 (1–10)	1 (12%)	7 (88%)	100%	6 (75%)	Patients with hypoechoic, intratesticular masses measuring 1 cm or less.	[46]
Tuygun et al. (2014)	10	37	17.5 (10–20)	4 (40%)	6 (60%)	100%	0	No paratesticular lesions, size of the lesion smaller than 20 mm and no known presence of elevated tumor markers or metastatic disease.	[58]
Xiao et al. (2019)	158	45.4	47.2	130 (82%)	28 (18%)	NA	23 (15%)	NA	[59]

N = number; FSE = frozen section examination; TSS = testis sparing surgery; NA = not available. * Some patients were followed on US only, and lesions were stable on US.

## Data Availability

Not applicable.

## Supplementary Materials

The following supporting information can be downloaded at: https://www.mdpi.com/article/10.3390/jcm11195770/s1, PROSPERO Protocol; Electronic Search Strategy; Table S1: List of articles included in this systematic review.
