# Supplementary material for: Prevalence and Management of Incidental Testicular Masses—A Systematic Review
Source: J Clin Med. 2022 Sep 29;11(19):5770. doi: 10.3390/jcm11195770 (PMC9573452; doi:10.3390/jcm11195770)
Supplement: Supplementary file 1 [file jcm-11-05770-s001.zip › jcm-1873536-supplementary.pdf]

Supplementary data:

I. PROSPERO Protocol

Protocol "Incidental testicular masses. Prevalence and management: systematic review"

Authors: Daniel Henriques[1] (https://orcid.org/0000-0002-5855-5979); Ricardo Leão[1] (orcid.org/0000-0003-3719-717X); Anabela Mota Pinto[1] (https://orcid.org/0000-0002-0820-9568); Helena Donato[2] (https://orcid.org/0000-0002-1905-1268)

[1] Faculdade de Medicina da Universidade de Coimbra, Coimbra – Portugal

[2] Documentation and Scientific Information Service, Centro Hospitalar e Universitário de Coimbra, Coimbra – Portugal.

Funding sources/sponsors: None.

Conflicts of interest: None.

Condition or domain being studied:

Incidental identification of asymptomatic testicular masses of small dimensions is increasingly frequent, due to the massification of testicular ultrasonography use for other indications, especially in the study of male infertility. The approach to these patients is controversial, due to the difficulty to ascertain the malignant or benign nature of these lesions without resorting to invasive methods or surgery.

Due to the fear of malignancy, the approach to these growths is currently radical orchiectomy. However, it is estimated that the prevalence of malignant lesions in this context is low, which suggests the reasonability to opt for a less radical approach.

In order to select the patients eligible, several factors that suggest an increased risk of developing a malignant testicular tumour have been described (cryptorchidism, infertility, contralateral tumour and family history) and therefore can contribute to justify the option for a less conservative approach. Some analytical resources, such as tumour markers or imagiological features, nominately the mass' size, vascularization, and microcalcifications, may also suggest the likelihood of malignancy.

In recent studies, the validity of conservative surgery with frozen section examination, or even active surveillance, is explored as sensate alternatives for approaching a group of selected patients with incidental testicular masses, presenting promising results.

Participants/population:

Adults (18 years) presenting with an impalpable testicular mass incidentally diagnosed by ultrasonography.

Intervention(s), exposure(s):

Patients submitted to a conservative approach, nominately active surveillance (resorting to testicular ultrasonography) or conservative surgery associated with frozen section examination.

Comparator(s)/control:

Radical orchiectomy.

Review question.

Main questions:

1. Is observation an acceptable option for the management of small testicular masses?

2. How does conservative surgery associated with frozen section examination compare to radical orchiectomy in the management of incidental testicular masses?

Secondary questions:

3. What is the prevalence of incidental testicular masses in populations of men studied by testicular ultrasound or other means?

4. What proportion of incidental masses exhibit malignant behaviour/pathology in FSE or orchiectomy?

5. Should small (which size) testicular masses always be managed with testis sparing surgery?

6. What are the main risk factors and predictive characteristics for malignancy of these incidental findings?

P – Small testicular masses

I – Testicular Sparing Surgery

C – Observation/Radical Orchiectomy

O – Cancer (disease)/progression

Searches:

We will identify studies from the following databases: PubMed, EMBASE (via Elsevier), Cochrane Central Register of Controlled Trials (CENTRAL): Cochrane Central Register of Controlled Trials (via Wiley Online Library), Web of Science (via Clarivate Analytics), ClinicalTrials.gov, MedRxiv.

We will search the mentioned databases from September 2020.

There are no geographic restrictions. We will only include studies in English language.

Search in each database will be performed from inception to September 2020. Additional end searches of the reference lists of all included studies will be conducted to ensure completeness of the search.

The search strategy will be developed in consultation with a medical librarian with expertise in systematic review searching.

A variety of terms related to key subject areas of review question will be used. Keywords or database specific subject headings (Eg: MeSH, and boolean operators (OR) and (AND) will be used to combine search terms. The search terms will be adjusted to the specificities of the different databases.

Types of study to be included:

Prospective cohort studies, randomised controlled trials, cross-sectional studies, case-control studies, case series or conference proceedings will be included.

Data extraction (selection and coding):

Two independent reviewers will extract relevant data from each selected study, using a standardized data extraction electronic form and, when applicable, using one of the available support checklists: Centre for Evidence-Based Medicine (CEBM), Cochrane Collaboration, Critical Appraisal Skills Programme (CASP). Any discrepancies in data extraction will be resolved by consensus or discussion with a third reviewer if needed.

The extracted data will consist of: 1. General information (date; who performed data extraction); 2. Study identification (authors, title, year of publication, volume, issue and pages); 3. Study characteristics (study setting, objectives, study design, sample size, inclusion and exclusion criteria); recruitment methodology – e.g. randomization, retrospective or prospective cohort -, controls, follow-up length, dropout rate, source of funding, country of origin); 4. Participants' characteristics (age, morbidities; reason for testicular imaging study); 5. Variables that could influence outcomes (age, lesion size, lesion imagiological characteristics, smoking, infertility, history of cryptorchidism, testicular atrophy, history of testicular tumour, history of /contralateral tumour). 6. Outcomes (malignant histology on pathology); 7. Effect size for associations reported between the identified variables and outcomes.

Only the information which is relevant to this systematic review research question will be extracted; if there are missing data about study characteristics, methods, variables, outcomes or measures of association, we may consider contacting the study authors asking for any available data. If the same data have been reported in multiple study publications, the duplicates will be deleted, to minimise the overrating of any variable or outcome investigated in the same sample.

Risk of bias (quality) assessment:

The risk of bias of each included study will be assessed by two review authors working independently. Any disagreements will be resolved by discussion or by consulting a third review author. Risk of bias will be assessed by using the QUIPS tool as recommended by the Cochrane Prognosis Methods Group.

Strategy for data synthesis:

Meta-analyses will be performed if valid data are available to assess the association between a clinical feature and the risk of testicular cancer, or a given therapeutic option and its outcome if sufficiently homogeneous (subgroups of) studies. We define sufficiently homogeneous subgroups as being those in which there is a similar population and the estimates of effects have been reported using a common measure (i.e all HR, or all OR). We will conduct separate meta-analyses of the different measures of association (OR, HR) as appropriate, both unadjusted and adjusted for other factors if the data permit. Meta-analyses will be conducted in RevMan 5.3 10. Meta-analyses will be summarised with pooled estimates of the effect size and 95% CIs, estimates of σ^2 (between-study variance) and 95% prediction intervals for the prognostic effect in a single population.

Analysis of subgroups or subsets:

We define sufficiently homogeneous subgroups as being those in which there is a similar population and the estimates of effects have been reported using a common measure (i.e all HR, or all OR).

Main outcome(s):

1. Risk factors for cancer amongst small testicular masses

Additional outcome(s):

2. Prevalence of small testicular masses

3. Histology of small testicular masses (malignant vs benign)

4. Predictive value of Frozen Sections

5. Rate of disease recurrence in testis sparing approaches

Keywords: testicular neoplasms; incidental findings; orchiectomy; frozen sections; risk factors

II. Electronic Search Strategy

The electronic search strategy used on PubMed database is described below:

Search number,Query,Sort By,Filters,Search Details,Results,Time

1,((testis OR testicular OR testicle OR seminomatous OR nonseminomatous) AND (cancer OR carcinoma OR neoplasm OR malignancy OR tumor OR tumour OR mass OR incidental)) AND (frozen section OR partial orchidectomy OR partial orchiectomy OR sparing OR conservative OR active surveillance OR watch and wait OR watchful waiting) AND (enucleation OR orchidectomy OR orchiectomy OR radical OR castration),Most Recent,Humans,"(""teste""[All Fields] OR ""testi""[All Fields] OR ""testis""[MeSH Terms] OR ""testis""[All Fields] OR ""testes""[All Fields] OR ""inferior colliculi""[MeSH Terms] OR (""inferior""[All Fields] AND ""colliculi""[All Fields]) OR ""inferior colliculi""[All Fields] OR ""testicular""[All Fields] OR (""testis""[MeSH Terms] OR ""testis""[All Fields] OR ""testicle""[All Fields] OR ""testicles""[All Fields]) OR ""seminomatous""[All Fields] OR ""nonseminomatous""[All Fields]) AND (""cancer s""[All Fields] OR ""cancerated""[All Fields] OR ""canceration""[All Fields] OR ""cancerization""[All Fields] OR ""cancerized""[All Fields] OR ""cancerous""[All Fields] OR ""neoplasms""[MeSH Terms] OR ""neoplasms""[All Fields] OR ""cancer""[All Fields] OR ""cancers""[All Fields] OR (""carcinoma""[MeSH Terms] OR ""carcinoma""[All Fields] OR ""carcinomas""[All Fields] OR ""carcinoma s""[All Fields]) OR (""neoplasm s""[All Fields] OR ""neoplasms""[MeSH Terms] OR ""neoplasms""[All Fields] OR ""neoplasm""[All Fields]) OR (""malign""[All Fields] OR ""malignance""[All Fields] OR ""malignances""[All Fields] OR ""malignant""[All Fields] OR ""malignants""[All Fields] OR ""malignities""[All Fields] OR ""malignity""[All Fields] OR ""malignization""[All Fields] OR ""malignized""[All Fields] OR ""maligns""[All Fields] OR ""neoplasms""[MeSH Terms] OR ""neoplasms""[All Fields] OR ""malignancies""[All Fields] OR ""malignancy""[All Fields]) OR (""cysts""[MeSH Terms] OR ""cysts""[All Fields] OR ""cyst""[All Fields] OR ""neurofibroma""[MeSH Terms] OR ""neurofibroma""[All Fields] OR ""neurofibromas""[All Fields] OR ""tumor s""[All Fields] OR ""tumoral""[All Fields] OR ""tumorous""[All Fields] OR ""tumour""[All Fields] OR ""neoplasms""[MeSH Terms] OR ""neoplasms""[All Fields] OR ""tumor""[All Fields] OR ""tumour s""[All

Fields] OR ""tumoural""[All Fields] OR ""tumourous""[All Fields] OR ""tumours""[All Fields] OR ""tumors""[All Fields]) OR (""cysts""[MeSH Terms] OR ""cysts""[All Fields] OR ""cyst""[All Fields] OR ""neurofibroma""[MeSH Terms] OR ""neurofibroma""[All Fields] OR ""neurofibromas""[All Fields] OR ""tumor s""[All Fields] OR ""tumoral""[All Fields] OR ""tumorous""[All Fields] OR ""tumour""[All Fields] OR ""neoplasms""[MeSH Terms] OR ""neoplasms""[All Fields] OR ""tumor""[All Fields] OR ""tumour s""[All Fields] OR ""tumoural""[All Fields] OR ""tumourous""[All Fields] OR ""tumours""[All Fields] OR ""tumors""[All Fields]) OR (""molecular weight""[MeSH Terms] OR (""molecular""[All Fields] AND ""weight""[All Fields]) OR ""molecular weight""[All Fields] OR ""mass""[All Fields]) OR (""incidental""[All Fields] OR ""incidentally""[All Fields] OR ""incidentals""[All Fields])) AND (""frozen sections""[MeSH Terms] OR (""frozen""[All Fields] AND ""sections""[All Fields]) OR ""frozen sections""[All Fields] OR (""frozen""[All Fields] AND ""section""[All Fields]) OR ""frozen section""[All Fields] OR ""cryoultramicrotomy""[MeSH Terms] OR ""cryoultramicrotomy""[All Fields] OR (""frozen""[All Fields] AND ""section""[All Fields]) OR ((""partial""[All Fields] OR ""partials""[All Fields]) AND (""orchiectomy""[MeSH Terms] OR ""orchiectomy""[All Fields] OR ""orchidectomies""[All Fields] OR ""orchidectomy""[All Fields])) OR ((""partial""[All Fields] OR ""partials""[All Fields]) AND (""orchiectomy""[MeSH Terms] OR ""orchiectomy""[All Fields] OR ""orchiectomies""[All Fields])) OR (""spare""[All Fields] OR ""spared""[All Fields] OR ""spares""[All Fields] OR ""sparing""[All Fields]) OR (""conservancies""[All Fields] OR ""conservancy""[All Fields] OR ""conservancy s""[All Fields] OR ""conservation""[All Fields] OR ""conservational""[All Fields] OR ""conservations""[All Fields] OR ""conservative""[All Fields] OR ""conservatively""[All Fields] OR ""conservatives""[All Fields] OR ""conserve""[All Fields] OR ""conserved""[All Fields] OR ""conserves""[All Fields] OR ""conserving""[All Fields]) OR (""watchful waiting""[MeSH Terms] OR (""watchful""[All Fields] AND ""waiting""[All Fields]) OR ""watchful waiting""[All Fields] OR (""active""[All Fields] AND ""surveillance""[All Fields]) OR ""active surveillance""[All Fields]) OR ((""watch""[All Fields] OR ""watched""[All Fields] OR ""watches""[All Fields] OR ""watching""[All Fields]) AND ""wait""[All Fields]) OR (""watchful waiting""[MeSH Terms] OR (""watchful""[All Fields] AND ""waiting""[All Fields]) OR ""watchful waiting""[All Fields])) AND (""enuclation""[All Fields] OR ""enucleate""[All Fields] OR ""enucleated""[All Fields] OR ""enucleates""[All Fields] OR ""enucleating""[All Fields] OR ""enucleation""[All Fields] OR ""enucleations""[All Fields] OR ""enucleative""[All Fields] OR (""orchiectomy""[MeSH Terms] OR ""orchiectomy""[All Fields] OR ""orchidectomies""[All Fields] OR ""orchidectomy""[All Fields]) OR (""orchiectomy""[MeSH Terms] OR ""orchiectomy""[All Fields] OR ""orchiectomies""[All Fields]) OR (""radical""[All Fields] OR ""radical s""[All Fields] OR ""radicals""[All Fields]) OR (""castrate""[All Fields] OR ""castrated""[All Fields] OR ""castrates""[All Fields] OR ""castrating""[All Fields] OR ""castration""[MeSH Terms] OR ""castration""[All Fields] OR ""castrations""[All Fields] OR ""castrator""[All Fields] OR ""castrators""[All Fields] OR ""orchiectomy""[MeSH Terms] OR ""orchiectomy""[All Fields]))",743,13:07:46

III. Supplementary Table S1 – List of articles included in this systematic review.

Author (year)	Title	References
Ates et al. (2016)	Testis-sparing surgery in small testicular masses not suspected to be malignant	8
Avci et al. (2008)	Nine cases of nonpalpable testicular mass: an incidental finding in a large scale ultrasonography survey	9
Ayati et al. (2014)	Management of nonpalpable incidental testicular masses: experience with 10 cases	47
Benelli et al (2017)	Evaluation of the decision-making process in the conservative approach to small testicular masses	10
Bieniek et al. (2017)	Prevalence and management of incidental small testicular masses discovered on ultrasonographic evaluation of male infertility	11

Author (year)	Title	References
Bojanic et al. (2017)	Testis sparing surgery for treatment of small testicular lesions: is it feasible even in germ cell tumors?	12
Bozzini et al. (2014)	Role of frozen section examination in the management of testicular nodules: a useful procedure to identify benign lesions	48
Browne et al. (2003)	Intra-operative ultrasound-guided needle localization for impalpable testicular lesions	13
Buckspan et al. (1989)	Intraoperative ultrasound in the conservative resection of testicular neoplasms	14
Carmignani et al. (2003)	High incidence of benign testicular neoplasms diagnosed by ultrasound	15
Carmignani et al. (2004)	Detection of testicular ultrasonographic lesions in severe male infertility	16
Colpi et al (2005)	Testicular-sparing microsurgery for suspected testicular masses	17
Comiter et al. (1995)	Nonpalpable intratesticular masses detected sonographically	18
Connolly et al. (2006)	Value of frozen section analysis with suspected testicular malignancy	49
Connolly et al. (2006)	Carefully selected intratesticular lesions can be safely managed with serial ultrasonography	19
Corrie et al. (1991)	Management of ultrasonically detected nonpalpable testis masses	20
Csapo et al. (1988)	Impalpable testicular tumors diagnosed by scrotal ultrasonography	21
De Stefani et al. (2012)	Microsurgical testis-sparing surgery in small testicular masses: seven years retrospective management and results	22
Dell'Atti (2016)	Efficacy of ultrasound-guided testicle-sparing surgery for small testicular masses	50
Dell'Atti et al. (2018)	Are ultrasonographic measurements a reliable parameter to choose non-palpable testicular masses amenable to treatment with sparing surgery?	51
Eifler et al. (2008)	Incidental testicular lesions found during infertility evaluation are usually benign and may be managed conservatively	23
Fabiani et al. (2014)	Diagnostic ultrasound-guided excisional testicular biopsy for small (<1cm) incidental nodules.A single institution experience	24
Ferretti et al. (2014)	Testicular-sparing surgery for bilateral or monorchide testicular tumours: a multicenter study of long-term oncological and functional results	52
Galosi et al. (2016)	Testicular sparing surgery in small testis masses: A multinstitutional experience	53
Gentile et al. (2013)	Can testis-sparing surgery for small testicular masses be considered a valid alternative to radical orchiectomy? A prospective single-center study	25
Haas et al. (1986)	The high incidence of benign testicular tumors	5

Author (year)	Title	References
Hallak et al. (2009)	Organ-sparing microsurgical resection of incidental testicular tumors plus microdissection for sperm extraction and cryopreservation in azoospermic patients	26
Hindley et al. (2003)	Impalpable testis cancer	27
Hopps and Goldstein (2002)	Ultrasound guided needle localization and microsurgical exploration for incidental nonpalpable testicular tumors	28
Horstman et al. (1994)	Management of testicular masses incidentally discovered by ultrasound	29
Isidori et al. (2014)	Differential diagnosis of nonpalpable testicular lesions: qualitative and quantitative contrast-enhanced US of benign and malignant testicular tumors	30
Khan et al. (2018)	Testis sparing surgery for small testicular masses and frozen section assessment	31
Kizilay et al. (2019)	Long-term results of patients with testicular tumors undergoing testis sparing surgery: a single-center experience	32
Lagabrielle et al. (2018)	Testicular tumours discovered during infertility workup are predominantly benign and could initially be managed by sparing surgery	33
Leonhartsberger et al. (2014)	Organ preservation technique without ischemia in patients with testicular tumor	34
Leroy et al. (2003)	Value of frozen section examination for the management of nonpalpable incidental testicular tumors	35
Li et al. (2017)	The value of active ultrasound surveillance for patients with small testicular lesions	60
Matei et al. (2017)	Reliability of frozen section examination in a large cohort of testicular masses: what did we learn?	54
Muller et al. (2006)	Management of incidental impalpable intratesticular masses of ≤ 5 mm in diameter	36
Onur et al. (2008)	Scrotal ultrasonography: should it be used in routine evaluation of infertile men?	37
Passarella et al. (2003)	Testicular-sparing surgery: a reasonable option in selected patients with testicular lesions	55
Pierik et al. (1999)	Is routine scrotal ultrasound advantageous in infertile men?	38
Powell and Tarter (2006)	Management of nonpalpable incidental testicular masses	39
Rolle et al. (2006)	Microsurgical testis-sparing surgery for nonpalpable hypoechoic testicular lesions	40
Sakamoto et al. (2006)	Color doppler ultrasonography as a routine clinical examination in male infertility	41
Scandura et al. (2018)	Incidentally detected testicular lesions <10 mm in diameter: can orchidectomy be avoided?	61

Author (year)	Title	References
Sheynkin et al. (2004)	Management of nonpalpable testicular tumors	42
Shilo et al. (2012)	Testicular sparing surgery for small masses	43
Shilo et al. (2012)	The predominance of benign histology in small testicular masses	6
Shtricker et al. (2015)	The value of testicular ultrasound in the prediction of the type and size of testicular tumors	62
Silverio et al. (2015)	Fourteen-year experience with the intraoperative frozen section examination of testicular lesion in a tertiary university center	56
Tackett et al. (1986)	High resolution sonography in diagnosing testicular neoplasms: clinical significance of false positive scans	44
Tal et al. (2004)	Incidental testicular tumors in infertile men	45
Tokuc et al. (1992)	Accuracy of frozen section examination of testicular tumors	57
Toren et al. (2010)	Small incidentally discovered testicular masses in infertile men- is active surveillance the new standard of care?	46
Tuygun et al. (2014)	Evaluation of frozen section results in patients who have suspected testicular masses: a preliminary report	58
Xiao et al. (2019)	Radical and testis-sparing surgery for primary testicular tumors: A single-center experience	59
